# Influence of environmental factors and body condition on the post-oviposition behavior in the emerald glass frog *Espadarana prosoblepon* (Centrolenidae)

**DOI:** 10.7717/peerj.13616

**Published:** 2022-06-16

**Authors:** Johana Goyes Vallejos, Abner D. Hernández-Figueroa

**Affiliations:** 1 Division of Biological Sciences, University of Missouri - Columbia, Columbia, MO, United States of America; 2 Environmental Sciences Department, University of Puerto Rico –Rio Piedras, San Juan, Puerto Rico

**Keywords:** Anuran, Costa Rica, Egg clutch, Reproductive biology, Egg attendance

## Abstract

In species with parental care behaviors, parents may adjust the intensity and duration of their care if fluctuation in factors such as environmental variables or body condition affects offspring survival. In the face of environmental changes, many egg-laying species remain with their clutch for extended periods if this behavioral adjustment provides tangible benefits to the offspring. However, the length of time parents stay with the offspring may also differ depending on the individual’s body condition. In the glass frog family (Centrolenidae), several species exhibit long-term egg attendance in which they remain with their clutch for several days after oviposition takes place. For some of them, changes in environmental variables lead to increased parental care efforts. For the species in which parents remain with their offspring for a short period (less than 24 hours), it is less clear if this constitutes parenting behavior, and whether parents adjust their efforts as a function of environmental change or the parent’s body condition remains unexplored. We studied a population of the Emerald Glass Frog, *Espadarana prosoblepon*, a species that exhibits a short period of quiescence after oviposition (less than three hours). Our study aimed to determine whether females alter the length of their post-oviposition quiescence period in response to changes in environmental variables (i.e., temperature, humidity, rainfall, and mean wind speed) or female body condition. Pairs in amplexus were captured in the field and transported to semi-natural enclosures to record the duration of post-oviposition quiescence using infrared cameras. Females’ post-oviposition quiescence lasted an average of 67.4 ± 26.6 min (range = 22.7–158.3 min). We did not find a significant relationship between the duration of the post-oviposition quiescence and any of the environmental variables tested. Similarly, post-oviposition quiescence duration was not influenced by female body condition. Because the variation observed in the duration of post-oviposition quiescence was not related to changes in extrinsic (environmental) or intrinsic (body condition) factors, we found no evidence that females of *E. prosoblepon* modify their post-oviposition behavior in response to any of the variables examined in this study. Future research investigating the adaptive significance of the post-oviposition quiescence observed in this species is needed to understand how this behavior is related to parental care efforts.

## Introduction

Many egg-laying species exhibit parental care behavior by remaining with their offspring after oviposition occurs, a behavior usually defined as “egg attendance”. This behavior collectively observed in birds, for example, may entail tending to the nest by protecting it from predators and sitting on top of the eggs until they hatch. Species of reptiles, fish, amphibians and some invertebrates also exhibit egg attendance behaviors. If egg attendance increases offspring survival, then this behavior can be considered parental care (Smiseth, Kölliker & Royle, 2014). In addition, parents may exhibit behavioral plasticity by increasing or decreasing the amount of time they spend tending to their offspring in response to abiotic or biotic factors to increase the offspring’s chances of survival (Royle, Russell & Wilson, 2014). For example, in species where eggs are susceptible to fluctuations in weather conditions, changes in temperature and relative humidity can increase the rate of evaporative water loss. Thus, parents might prevent desiccation by covering the clutch with their whole body to reduce surface exposure or by increasing the duration and frequency of brooding bouts. As a result, extended bouts of care may ultimately reduce the risk of desiccation and mortality (Delia, Ramirez-Bautista & Summers, 2013; Lourdais, Hoffman & DeNardo, 2007; Taigen, Pough & Stewart, 1985). Likewise, parents may adjust the duration of care depending on the quantity of resources acquired before egg-laying occurs or their current body condition (Aldrich & Raveling, 1983; Dubiec, 2011).

In amphibians, parental care has been observed in 10–20% of species, with egg attendance being the most common form of care (Crump, 1996; Furness & Capellini, 2019; Schulte et al., 2020). Here, we define egg attendance as the behavior exhibited by either of the parents in which they remain with the eggs at the oviposition site (Crump, 1996). Parents can provide different levels of care: from protection against predators (also known as egg guarding) to jostling of the eggs to prevent developmental abnormalities or protection from harsh environmental conditions and pathogens (Bickford, 2002; Bickford, 2004; Burrowes, 2000; Cheng & Kam, 2010; Consolmagno et al., 2016; Delia, Ramírez-Bautista & Summers, 2014; Poo & Bickford, 2013). In the glass frog family (Anura: Centrolenidae), several species exhibit egg attendance behavior, and the benefits of long-term attendance have been observed. For example, parents may protect the egg clutch by actively fending off predators or providing hydration through direct contact of the ventral pouch with the eggs or by urinating on them, ultimately resulting in increased embryonic survivorship (Vockenhuber, Hodl & Amézquita, 2009; Bravo Valencia & Delia, 2016; Delia, Bravo-Valencia & Warkentin, 2017; Salgado & Guayasamin, 2018).

Moreover, egg attendance behavior might be sensitive to fluctuations in environmental variables resulting in more prolonged bouts of care. Increases in the frequency of egg attendance directly affect offspring survival when environmental conditions are suboptimal (Delia, Ramirez-Bautista & Summers, 2013). However, in species with so-called “first-night care” (or short-term attendance; <1 day), performed exclusively by females—a behavior that appears to be the ancestral condition for the family—the benefits are not as clear (Delia, Bravo-Valencia & Warkentin, 2017), and the amount of information about their reproductive behavior is sparse and primarily anecdotal (but see Delia, Bravo-Valencia & Warkentin, 2017). Studies on other species of glass frogs believed to have “first-night care”, such as members of the genus *Teratohyla* (Díaz-Ricaurte, Guevara-Molina & Serrano, 2019), *Nymphargus grandisonae* (Guevara-Molina & Vargas-Salinas, 2014), and *Centrolene lynchi* (Dautel et al., 2011) did not systematically examine if environmental factors influence how long mothers remain with their clutch, a potential indicator of an adaptive parental response to changes in the environment (Delia, Ramirez-Bautista & Summers, 2013; Royle, Russell & Wilson, 2014).

*Espadarana prosoblepon*, a glass frog abundantly found from eastern Honduras to Panama, and along the Pacific slopes of the Andes of Colombia and Ecuador at elevations ranging from 20 to 1900 m above sea level (m.a.s.l.; Savage, 2002; Guayasamin et al., 2020), is one of the species within the family in which females stay with the egg clutch for a few hours after oviposition (Jacobson, 1985; Delia, Bravo-Valencia & Warkentin, 2017). In this species, males initiate amplexus by climbing on the back of an approaching female, and then, the amplectant female will move throughout the vegetation looking for a suitable oviposition site. Males retire onto the vegetation as soon as egg-laying and fertilization end, occasionally vocalizing as they move away from the oviposition site (Savage, 2002). Conversely, previous studies have shown that females remain with the clutches in a period of “quiescence”; some females remain as little as 10 min while others remain as long as three hours (Jacobson, 1985; Basto-Riascos, López-Caro & Vargas-Salinas, 2017). However, studies exploring the function and consequences of variation in this female behavior are scarce. Thus, it remains uncertain whether this behavior, termed “egg attendance” or “brooding” in previous studies (Jacobson, 1985; Basto-Riascos, López-Caro & Vargas-Salinas, 2017; Delia, Bravo-Valencia & Warkentin, 2017), increases offspring survival; therefore, we refrain from using either of these terms. Here, we define the behavior of females of *E. prosoblepon* after fertilization and oviposition have occurred and by which females remain with their egg clutches as “post-oviposition quiescence.”

*Espadarana prosoblepon* inhabits multiple habitat types (*i.e.,* lowland forests, rainforests, lower montane forests, and urban areas), which differ in environmental parameters. Thus, *E. prosoblepon* is a particularly well-suited species to investigate the impact of environmental factors on the duration of post-oviposition quiescence. In this study, we examined the variation in the duration of the post-oviposition quiescence period among females of *E. prosoblepon* and its response to environmental and energetic conditions. Specifically, we asked if environmental variables and body condition influence the amount of time *E. prosoblepon* females remain with the clutches immediately after oviposition, as a first step to determine the function of this behavior. To answer these questions, we used enclosures subject to natural environmental conditions in the field to closely monitor the pre-and post-oviposition behavior of pairs of *E. prosoblepon* in southwestern Costa Rica. No studies have examined whether females of this species adjust their post-oviposition quiescence duration with changing environmental conditions or if such variation is correlated with female body condition, interpreted as an estimate of the energetic state of an individual (*i.e.,* relative fat reserves; Jakob, Marshall & Uetz, 1996).

We addressed the following questions: (1) How much variation in the duration of the post-oviposition quiescence period exists among *E. prosoblepon* females? (2) Is the duration of the post-oviposition quiescence period influenced by environmental variables, such as temperature, wind speed, humidity, and amount of rainfall? (3) Is the duration of the post-oviposition quiescence period affected by female body condition?

We hypothesized that females would remain in post-oviposition quiescence for extended periods when environmental conditions may decrease the likelihood of offspring survival: when humidity and rainfall are lower (negative relationship) and when temperature and wind speed are higher (positive relationship). Body condition might also affect the duration of the post-oviposition quiescence period independently of environmental conditions. The amount of time a female can remain with the clutch after oviposition might be state-dependent, and females in better condition may be able to remain with the clutches for longer after oviposition, especially if this provides a benefit to the offspring. Conversely, females might need a resting period after searching for suitable oviposition sites and egg-laying, and thus females in poorer condition might remain in quiescence for longer periods of time. Lastly, we also describe other aspects of the reproductive biology and post-oviposition behavior of *E. prosoblepon*, building upon the knowledge gained by previous studies of the reproductive biology in this species.

## Materials & Methods

### Study site and visual encounter surveys

This study took place between June 8 and July 23, 2019, at Las Cruces Biological Station, Puntarenas Province, Costa Rica (8.786°N, 82.959°W datum = WGS84, 1100 m.a.s.l.). Our sampling period corresponded to the beginning of the rainy season (May–December), which has an average annual rainfall of 4000 mm (data from Las Cruces weather station). Our study site encompassed an area of approximately 500 m^2^ at the edge of the station’s botanical garden. We surveyed the area surrounding a small stream (“Culvert Creek”), where the predominant vegetation is palm trees, gingers, and giant ferns. We conducted surveys between 1900–2300 h at night to locate pairs of *E. prosoblepon* in amplexus. After finding pairs in amplexus, we marked their location and transferred them into an outdoor enclosure placed within our study area. The enclosure had four mesh cages adjacent to one another (dimensions: 38 × 50 × 75 cm; Fig. S1). The breathable nature of the mesh surrounding the enclosure allowed for the environmental conditions inside the cages to vary according to the outdoor conditions. We used plastic bags to transport the amplectant pairs to their respective enclosures (one pair per cage). Pairs stayed in amplexus during transportation and after being transferred to the cages. After capture and subsequent transport, pairs remained in amplexus for an average of 5.8 ± 1.4 h (range = 3.6–7.8 h) before oviposition occurred. We minimized variation due to differences in oviposition substrate by providing a fern leaf of the same species hung from a bamboo post in each cage. The bamboo post doubled as a refuge for the frogs. Each cage also contained leaf litter and a water bowl.

### Behavioral observations and measurements

We recorded time-lapse videos of the amplectant pair’s behavior in 0.5 s intervals from approximately 2300 h until 0600 h of the next day by placing an infrared (IR) video camera (GoPro Hero5 Black with a modified IR lens) with an external IR illuminator on the opposite corner of the cage. The pairs were allowed to acclimate for at least 90 min before we started recording. Previous studies have shown that some species of glass frogs actively find dew on the vegetation and absorb it through their pelvic patch, presumably to posteriorly hydrate their clutches (Delia, Bravo-Valencia & Warkentin, 2017). Thus, cages were liberally sprayed manually with water at the start of sampling (ca. 1900 h) to provide this resource.

The resulting videos were used to obtain the exact time at which oviposition occurred. The duration of the post-oviposition quiescence period was defined as the length of time the female remained next to or covering the clutch with her body (partially or entirely) until she deserted it. The following night, we recorded the snout-vent length (SVL) and body mass of both females and males and marked them using Visible Implant Alpha Tags (Northwest Marine Technology, Inc.) to avoid sampling the same individuals twice. Individual frogs were released at the point of capture.

### Effect of environmental factors and body condition on the duration of the post-oviposition quiescence period

Meteorological and hydrological data were obtained from the Organization for Tropical Studies weather station at Las Cruces Biological Station. The weather station registers data at 15-min intervals. The values of temperature, mean wind speed and relative humidity at which oviposition occurred were based on the values of the corresponding 15-min interval at which fertilization and oviposition occurred. We used the maximum daily rainfall values of the day in which the pairs were found and transported to the enclosure.

All statistical analyses were performed using R version 3.6.2 (R Core Team, 2019). After checking the data for normality, we log-transformed the values of post-oviposition quiescence to approximate a normal distribution. To test the relationship between the length of post-oviposition quiescence and each of the environmental variables at the time of oviposition (*i.e.,* temperature (°C), mean wind speed (m/s), maximum daily rainfall (mm), and relative humidity (%)), we performed linear mixed models using the lmer() function from the ‘*lme4*’ package (Bates et al., 2015). First, we fitted four separate models for each environmental variable as a fixed effect and cage number as a random effect to account for differences among the cages within the enclosure. Mixed-effects models including pairwise interactions between environmental variables (six separate models) produced similar results. Multicollinearity was not an issue as none of the predictor variables showed strong correlations, in all cases with —*ρ*—<0.7. We used Akaike’s information criterion for small sample sizes (AIC_c_) model selection using the aictab() function in the ‘*AICcmodavg’* package (Mazerolle, 2020) to rank the ten models. We used AIC_c_ weights (*w*_*i*_) to evaluate model likelihood, considering a single best model *i* if *w*_i_ > 0.9 following Portet (2020). We also included in the model comparison a global model with all covariates as fixed effects and a null model (intercept-only model).

To assess the relationship between females’ body condition and the duration of the post-oviposition quiescence period, we fitted a mixed-effects linear model with the log-transformed values of post-oviposition quiescence as the response variable, Body Condition Index (BCI) as a covariate and cage number as a random effect. We calculated BCI using the residuals of the linear regression of the SVL and body mass (Schulte-Hostedde et al., 2005) using a linear regression. This “residual” body condition index satisfactorily controls for variation across body size, with positive values indicating “good” body condition and negative values indicating “poor” body condition (Jakob, Marshall & Uetz, 1996). Alpha was set at 0.05 in all tests. We present results as mean ± standard deviation (SD).

### Ethical note

All behavioral observations and field manipulations followed the Animal Behavior Society guidelines for the treatment of animals in behavioral research and teaching. Our study was approved by the Costa Rican Ministry of the Environment and Energy (MINAE) and the National System of Conservation Areas (SINAC) (approval number: R-SINAC-PNI-ACLAP-031-2019).

## Results

### Visual encounter surveys and natural history observations

We found 60 pairs in amplexus between 1900 h to 2200 h within our study area over the course of six weeks. Throughout our study, we recaptured seven males and four females. Four out of the seven males were recaptured one additional time. Recaptured females were found mating with the same male in all cases. The number of days between recaptures ranged from 1–25 days for the males and 15–21 days for the females. Two females and two males escaped before SVL, and mass measurements were taken, and one additional female escaped before being weighed. All recaptured individuals (females and males) were found near the vicinity of the original point of capture.

### Behavioral observations and measurements

**Figure 1:**
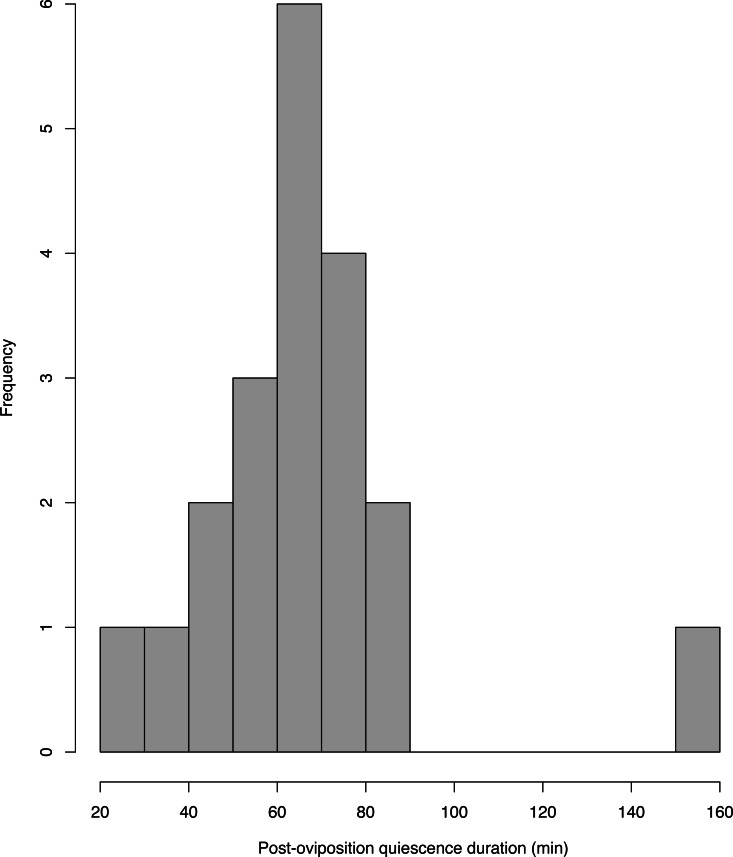
Histogram of the among-individual variation in the post-oviposition quiescence period in *E. prosoblepon* females. Most females remained in proximity of their clutch between 45–85 min (*n* = 20, mean = 68.5 min, SD = 26.9 min, range = 22.7–158.3 min).

The average male and female sizes were 23.3 ± 1.1 mm (range: 21.2–25.0, *n* = 58) and 25.6 ± 1.4 mm (range: 20.4–28.2, *n* = 58) respectively. On average, males weighed 0.7 ± 0.1 g (range: 0.6–0.8, *n* = 58), whereas females weighed 0.9 ± 0.1 g (range: 0.7–1.1, *n* = 57) after oviposition. A total of 47 amplectant pairs successfully laid their clutches on the surface of the fern frond provided in the cage; the remaining 13 pairs laid their clutches on the inner wall or on top of the leaf litter provided in the bottom of the cage, making it difficult to determine clutch size reliably. We only consider videos where the moment at which oviposition occurred and the time at which females deserted the clutch were unequivocal (Video S1), as some of the pairs laid their eggs behind the fronds’ pinnae or outside the camera frame. As a result, we obtained video footage of oviposition behavior in 20 pairs of *E. prosoblepon*. In our videos, it was not evident whether females “collect” dew from the substrate provided. None of the videos included in the analyses involved recaptured individuals. Immediately after fertilization, males leaped over the females’ heads onto the vegetation while females remained stationary after oviposition, covering approximately one-third of the clutch with their ventral sides. Females remained with the egg clutch between 22.7 and 158.3 min (mean ± SD = 67.4 ± 26.6 min, Fig. 1). Once the female deserted, neither of the parents returned to the clutch. The number of eggs per clutch ranged from 14–34 eggs (25.3 ± 4.7 eggs, *n* = 47). In no instances did females reposition themselves closer or on top of the egg clutch.

### Effect of environmental factors and body condition on the duration of the post-oviposition quiescence period

**Figure 2:**
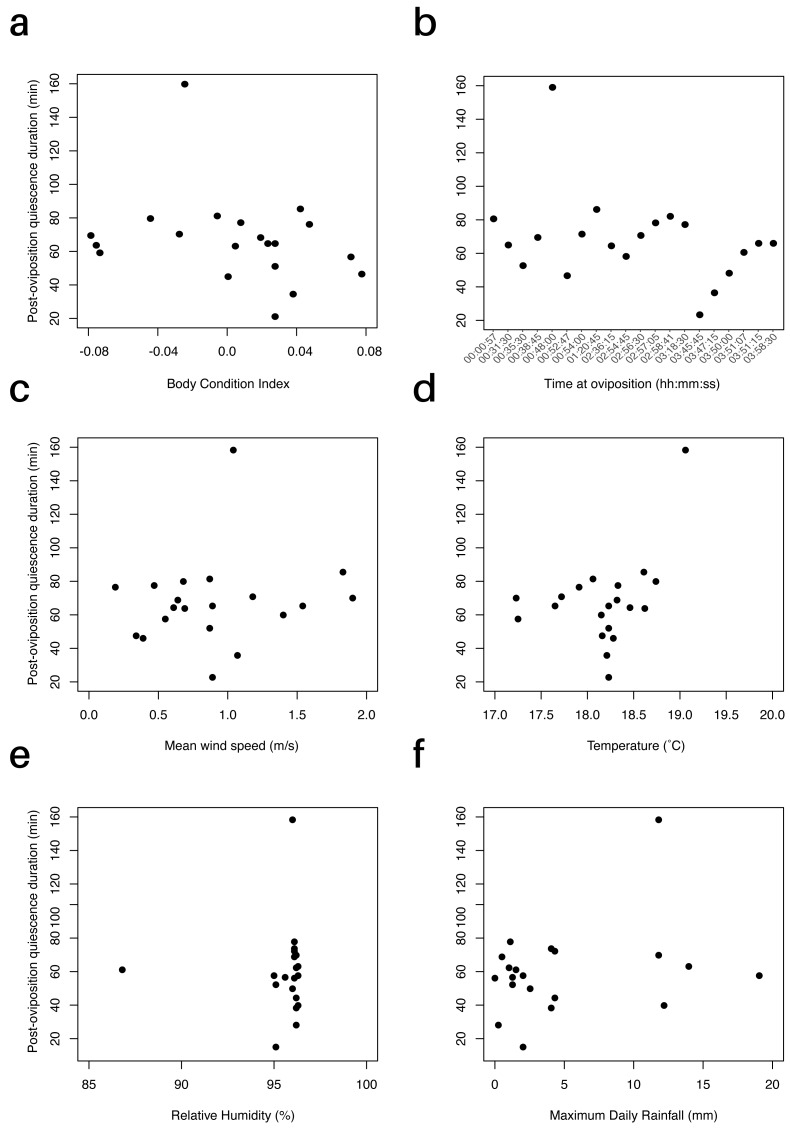
Effect of environmental factors and body condition on the duration of the post-oviposition quiescence period in *E. prosoblepon*. (A) Relationship between the duration of the post-oviposition quiescence period and female’s body condition index (BCI). BCI did not predict how long females remained with their clutch (*P* = 0.98). (B) Duration of the post-oviposition quiescence (min) over time at which oviposition occurred. Time at oviposition did not influence how long females stayed with their clutch (*P* = 0.21). (C–F) Relationship between duration of the post-oviposition quiescence period and the environmental variables measured. (C) There was no relationship between post-oviposition quiescence period (min) and mean wind speed, (D) temperature, (E) relative humidity, and (F) maximum rainfall (all *P* > 0.05). Maximum amount of rainfall was measured between 0700–2200 h.

We did not find a relationship between female body condition (BCI) and post-oviposition quiescence period (*P* = 0.98, Fig. 2A). Including clutch size in the linear regression yielded the same result (*P* = 0.74). The data point at 158.3 min represented an obvious outlier, but its removal (*post hoc*) did not change the results of this regression or subsequent regressions. We also evaluated if the time at which oviposition occurred influenced how long females stayed with their clutches; however, we did not find a significant effect (*F*_1,18_ = 1.664, *P* = 0.21). Oviposition occurred between 0057 h and 0358 h. Females that laid eggs earlier in the night did not exhibit longer post-oviposition quiescence periods (Fig. 2B).

Temperature and relative humidity at the time of oviposition for the 20 observed pairs ranged from 17.3 to 19.0 °C (18.2 ± 0.5 °C) and 86.4 to 96.4% (95.5 ± 2.1%), respectively. Maximum daily rainfall ranged from 0 to 19 mm (4.9 ± 5.5 mm), and mean wind speed ranged from 0.2 to 1.9 m/s (0.9 ± 0.5 m/s). None of these environmental variables had a significant relationship with the time females remained with their clutches (*P*_temp_ = 0.23, *P*_wind_ = 0.71, *P*_rain_ = 0.06, *P*_humidity_ = 0.99, Figs. 2C–2F). For the AIC_c_ model comparison, none of the models were supported by the experimental data (all *w*_*i*_ <0.34). Overall, including any of the environmental variables evaluated did not improve our ability to predict variation in the length of post-oviposition quiescence (Table S1).

## Discussion

Egg attendance is widespread among amphibians with parental care, yet our understanding of the factors contributing to variation in this behavior is limited. Within centrolenids, the time parents remain with their clutches immediately after fertilization is highly variable (Delia, Bravo-Valencia & Warkentin, 2017). However, we know little about the function of this behavior in species that remain with their clutches for less than a few hours. Our results suggest that environmental variables, time of oviposition, nor body condition index influenced the duration of the post-oviposition quiescence period, which we defined as the length of time females stayed with their clutches.

We predicted that females would remain with the clutches for shorter periods when rainfall and humidity were higher, as the risk of desiccation would be reduced. In another species of glass frog, *Hyalinobatrachium fleischmanni*, attendance behavior varies with changes in humidity and rainfall. In this species, males provide parental care, and they extend the duration of their care and the number of times they brood individual clutches when humidity decreases. Even a reduction in 2% relative humidity led to a significant increase in the proportion of time males spent with their clutches (Delia, Ramirez-Bautista & Summers, 2013). Similarly, we expected longer post-oviposition quiescence periods in *E. prosoblepon* with an increase in temperature or average wind speed if females prevent evaporation due to heat or wind by covering the clutch with their bodies (Lehtinen, Green & Pringle, 2014). However, this was not the case for our study population. It is important to consider that the environmental data were obtained at a larger scale, and thus, it is likely that the environmental data used in this study does not reflect the actual variation experienced at the microhabitat level. However, Delia, Ramirez-Bautista & Summers (2013) reported the values of temperature, relative humidity, and rainfall from a portable weather station along their study transect. They found strong evidence that males adjust their behavior in response to variation in these variables, which were not necessarily taken at the micro-scale for all monitored clutches. In addition, we note that the lack of significant effect in our models may be due to the narrow range of variation observed for some of the environmental variables tested (*i.e.,* relative humidity), which may not represent the actual variation at the microhabitat level. Nevertheless, our knowledge of how species with intricate post-oviposition behaviors respond to changes in microclimate continues to be limited. Therefore, temperature, relative humidity, and other variables at the microhabitat level are critical for understanding how environmental conditions influence post-oviposition behavior.

In most species of glass frogs where parents remain with the clutches for several days, removal experiments have shown that desiccation is the primary source of mortality in the absence of a caring parent, with extended periods of parental care (days) increasing offspring survival and hatching success (Bravo Valencia & Delia, 2016; Delia, Ramírez-Bautista & Summers, 2014; Lehtinen, 2004; Salgado & Guayasamin, 2018; Vockenhuber, Hodl & Amézquita, 2009). It is unknown whether females of *E. prosoblepon* can hydrate the clutches during the brief quiescence period after oviposition occurred and, if so, whether this “hydration effect” would be influenced by how long the mother stays with the clutch. Females may hydrate the eggs by emptying their bladder onto the egg clutch immediately after oviposition, thus, increasing the thickness of the jelly around the eggs. However, this egg hydration might depend more on the water capacity of the female’s bladder than the amount of time the female remains with the clutch. Clutches with a thin jelly layer are more susceptible to predation (Valencia-Aguilar et al., 2020); therefore, females may provide care to the embryos by creating a thick protective layer against desiccation and/or predators. Females of *E. prosoblepon* did not seem to collect water from the dew found on the vegetation; however, we acknowledge that this behavior might be difficult to detect from the video footage obtained, and thus whether females exhibit this behavior remains unknown.

A recent study in *Cochranella granulosa* and *Teratohyla pulverata*, two glass frog species in which the females remain with the egg clutches after oviposition between 90 to 170 min and 75 to 120 min, respectively, showed that females hydrate the clutch during this brief period and that this behavior reduces dehydration and the opportunity for predation by increasing the thickness of the jelly around the eggs. Likewise, parent removal experiments showed that clutches in which mothers were removed immediately after oviposition suffered higher levels of embryonic mortality, mainly due to dehydration (Delia, Bravo-Valencia & Warkentin, 2017). Delia, Bravo-Valencia & Warkentin (2017) demonstrated that the brief period of brooding exhibited by both *C. granulosa* and *T. pulverata* constitutes actual parental care in the form of egg attendance since clutches without their mothers had lower embryonic survival. It is not known if this is the case for *E. prosoblepon*, given that the adaptive benefits of the post-oviposition quiescence behavior have not been explored. In *E. prosoblepon*, most females remain with the clutches for less than two hours, and during the rainy season, clutches are typically exposed to rainfall a few hours after oviposition. Thus, variation in the duration of the post-oviposition quiescence period in this species might have a negligible hydration effect if clutches are exposed to rain throughout the season shortly after the females desert the clutch. In addition, *E. prosoblepon* arguably has a longer embryonic development period until hatching (20–25 days, J Goyes Vallejos, 2019, pers. obs.) compared to those observed in *C. granulosa* (13–17 days, Savage, 2002) and *T. pulverata* (11 days, Hawley, 2006). Therefore, the ratio between time expenditure with the clutch and number of days until hatching is lower for *E. prosoblepon*, than in *C. granulosa* and *T. pulverata*, indicating that females of *E. prosoblepon* remain with their clutches for a small proportion of time relative to the duration of embryonic development. Nevertheless, further studies should investigate this matter by experimentally demonstrating the benefits (or lack thereof) of the post-oviposition quiescence period using parent removal experiments. Therefore, adult removal experiments will be critical to answering this and other questions associated with the adaptive significance of the post-oviposition quiescence period in *E. prosoblepon*.

Currently, it is not clear how body condition affects parental care behavior patterns, particularly because, to the best of our knowledge, no studies have explored how body condition index might affect egg attendance duration in anuran species with brooding or egg attendance behavior. A study on the glass frog *Centrolene savagei* examined if male body size—but not body condition—increased embryonic survival, but they did not find a significant relationship (Ospina et al., 2019). In *E. prosoblepon*, we predicted that if longer periods of post-oviposition quiescence provide a benefit to the offspring, females in better body condition would be able to remain with their clutches for longer. As an alternative, females in poor condition (low body condition index) may benefit from a “resting period” after oviposition and remain for longer periods with the clutch. We did not find a relationship (positive or negative) between the duration of the post-oviposition quiescence period and female body condition; however, it remains a topic of interest in the field and should be explored further.

From our videos, we were able to ascertain the time when oviposition occurred and how long females stayed with the clutch. Most females (85%) remained with the clutch between 45–85 min (*n* = 17), although one female lasted 158.3 min. In this population, inter-individual variation in the duration of the post-oviposition quiescence period seems to be narrower than what Jacobson (1985) previously reported in Monteverde, Costa Rica (Table S2). The broader range in post-oviposition quiescence duration in Jacobson’s study might be attributed to observer disturbances during sampling. Still, it could also be the result of population and geographical differences. Notably, in a field study in an urban forest in the Central Andes of Colombia, *E. prosoblepon* females remained with the eggs between 45 to 175 min (Basto-Riascos, López-Caro & Vargas-Salinas, 2017); our findings were more consistent with these results. Together, this suggests that pairs in amplexus were not negatively affected by the semi-natural conditions of our outdoor enclosure, as all pairs successfully laid eggs and exhibited normal amplexus behavior with durations comparable to what has been observed in the wild (Jacobson, 1985; Basto-Riascos, López-Caro & Vargas-Salinas, 2017). However, differences in post-oviposition behavior across populations warrant further investigation considering the widespread distribution of *E. prosoblepon* in Central and South America; particularly those populations found in geographic locations with vastly different weather regimes in which temperature or other environmental factors are more extreme.

Observations of the oviposition and post-oviposition quiescence behavior in many centrolenids have increased in recent years (see Delia, Bravo-Valencia & Warkentin, 2017); however, for most species, intensive long-term field studies are needed to observe their natural behavior in the wild with minimal disturbance. In fact, in most cases, individuals are highly elusive, and the behavior of interest (*e.g.*, oviposition) is unpredictable. Furthermore, although observations in the wild are highly desirable for providing accurate quantitative estimates of natural history traits, they can be challenged by sample size limitations, especially in secretive species. Thus, observations in enclosures subject to natural environmental conditions may also prove useful, especially for recording large numbers of detailed observations in a controlled environment and allow—to some extent—researchers to control for confounding factors (Weygoldt, 1980; Gibson & Buley, 2004; Warkentin, Buckley & Metcalf, 2006a; Warkentin, Caldwell & McDaniel, 2006b; Oneto et al., 2010; Poo & Bickford, 2014; Salica, Vonesh & Warkentin, 2017; Spring et al., 2019).

Our mesh enclosures were subject to similar environmental conditions as individuals in the wild. We do not consider that there were significant negative impacts on the amplectant pairs as we took careful precautions during transportation and handling (including extensive acclimation time), and the observed pairs readily laid eggs on the provided substrate. All females remained with the clutches; however, in our videos, none of them repositioned themselves on top of the eggs (during or after oviposition), contrary to what has been observed in other species of glass frogs (Delia, Bravo-Valencia & Warkentin, 2017). Jacobson (1985) reported similar observations from the field, where more than half of the females just partially covered the egg clutch and lacked the “egg rotation” and brooding behavior observed in other species of glass frogs with egg attendance. Future studies should characterize the females’ post-oviposition behavior *in situ* to determine if there are real geographic differences in the duration of the post-oviposition quiescence period between this population at Las Cruces and what has been previously reported elsewhere (Basto-Riascos, López-Caro & Vargas-Salinas, 2017).

The mating system of *E. prosoblepon* has not been described in detail, but it has been broadly characterized as a resource-based mating system (Angeli et al., 2015). Females move for several meters before choosing an oviposition site (2–4 m, J Goyes Vallejos, 2021, pers. obs), and while they may lay their eggs inside a male’s calling territory, it is unclear how these territories constitute a resource. Here, we found that all our recaptured females mated with the same males in all instances. A similar observation in which a female mated twice with the same male has been reported for *Hyalinobatrachium cappellei*, a glass frog with prolonged male parental care (Valencia-Aguilar et al., 2020). Studies of animal mating systems commonly focus on the males’ reproductive success; hence, detailed information about females’ mating patterns is scarce. Exploring the mating patterns of males and females of *E. prosoblepon* was beyond the scope of this paper. However, we believe that these natural history observations may serve as a basis for future research on the *E. prosoblepon* mating system.

## Conclusions

In our study, we provided systematic observations of the post-oviposition behavior of *E. prosoblepon* females in semi-natural enclosures in the field. This way, we obtained reliable data on the time of oviposition and the duration of the post-oviposition quiescence period. Our results suggest that environmental variables do not influence females’ post-oviposition quiescence periods in this population found around a wet pre-montane forest in southern Costa Rica. Similarly, using BCI as a proxy, females’ body condition was not related to the duration of post-oviposition quiescence periods. Comparing these findings with other populations of *E. prosoblepon* throughout the species’ range will allow examination of how different environmental variables, geography, and amount of urbanization may affect different behavioral responses. Refining our knowledge on behavioral variation within Centrolenidae (male parental care, female parental care, and no care) is critical for understanding the patterns of parental care evolution in the family. We highlight the importance of testing predictions about the evolution of elaborated behaviors with natural history-informed studies. The gain and loss of egg attendance is equally probable (Furness & Capellini, 2019); and thus, natural history studies are imperative to elucidate the selection pressures (*i.e.,* costs and benefits of egg guarding and environmental factors) leading to the evolution of parental care behavior in this clade.

##  Supplemental Information

10.7717/peerj.13616/supp-1 Supplemental Information 1 Set up of the outdoor enclosure used to observe the post-oviposition behavior of *Espadarana prosoblepon* pairs in amplexus (A) Setup of the semi-natural enclosure. The enclosure had four partitions adjacent to each other (38 × 50 × 75 cm) walled with green mesh. (B) A pair of *Espadarana prosoblepon* moving through the provided substrate inside the enclosure after capture and transportation. (C) A clutch of *E. prosoblepon*’s eggs laid on the provided substrate.

10.7717/peerj.13616/supp-2 Supplemental Information 2 Model comparison table Summary of model comparison and Akaike information criterion (AIC_c_) values and Δ AIC_c_ values comparing different models examining the effect of environmental variables (temperature, humidity, mean wind speed, rainfall) on *Espadarana prosoblepon*’s post-oviposition behavior (POQ), using “cage number” as a random variable in all models.

10.7717/peerj.13616/supp-3 Supplemental Information 3 Summary of reproductive and behavioral biology parameters in *E. prosoblepon.* Comparison of reported values of female snout-vent length, amplexus duration, whether brooding behavior was observed, female coverage of the clutch, and the time females remained with their clutch (defined in this study as ”post-oviposition quiescence period”) across different studies. Table entries include “—” if not reported.

10.7717/peerj.13616/supp-4 Supplemental Information 4 *Espadarana prosoblepon* raw dataset: post-oviposition quiescence Information about clutch size, time at oviposition, snout-vent length and mass for both males and females, post-oviposition quiescence duration, and environmental variables for 20 pairs for which videos were obtained.

## Additional Information and Declarations

### Data Availability

The following information was supplied regarding data availability: The raw data is available in the Supplemental Files. A time-lapse video showing oviposition and post-oviposition quiescence behavior in *Espadana prosoblepon* is available at figshare: Goyes Vallejos, Johana; Hernández-Figueroa, Abner D. (2022): Video S1. E_prosoblepon 2019.mp4. figshare. Media. https://doi.org/10.6084/m9.figshare.19516840.v1.
