# Supplementary material for: Influence of environmental factors and body condition on the post-oviposition behavior in the emerald glass frog Espadarana prosoblepon (Centrolenidae)
Source: PeerJ. 2022 Jun 16;10:e13616. doi: 10.7717/peerj.13616 (PMC9206843; doi:10.7717/peerj.13616)
Supplement: Supplemental Information 1 — (A) Setup of the semi-natural enclosure. The enclosure had four partitions adjacent to each other (38 × 50 × 75 cm) walled with green mesh. (B) A pair of Espadarana prosoblepon moving through the provided substrate inside the enclosure after capture and transportation. (C) A clutch of E. prosoblepon’s eggs laid on the provided substrate. [file peerj-10-13616-s001.pdf]

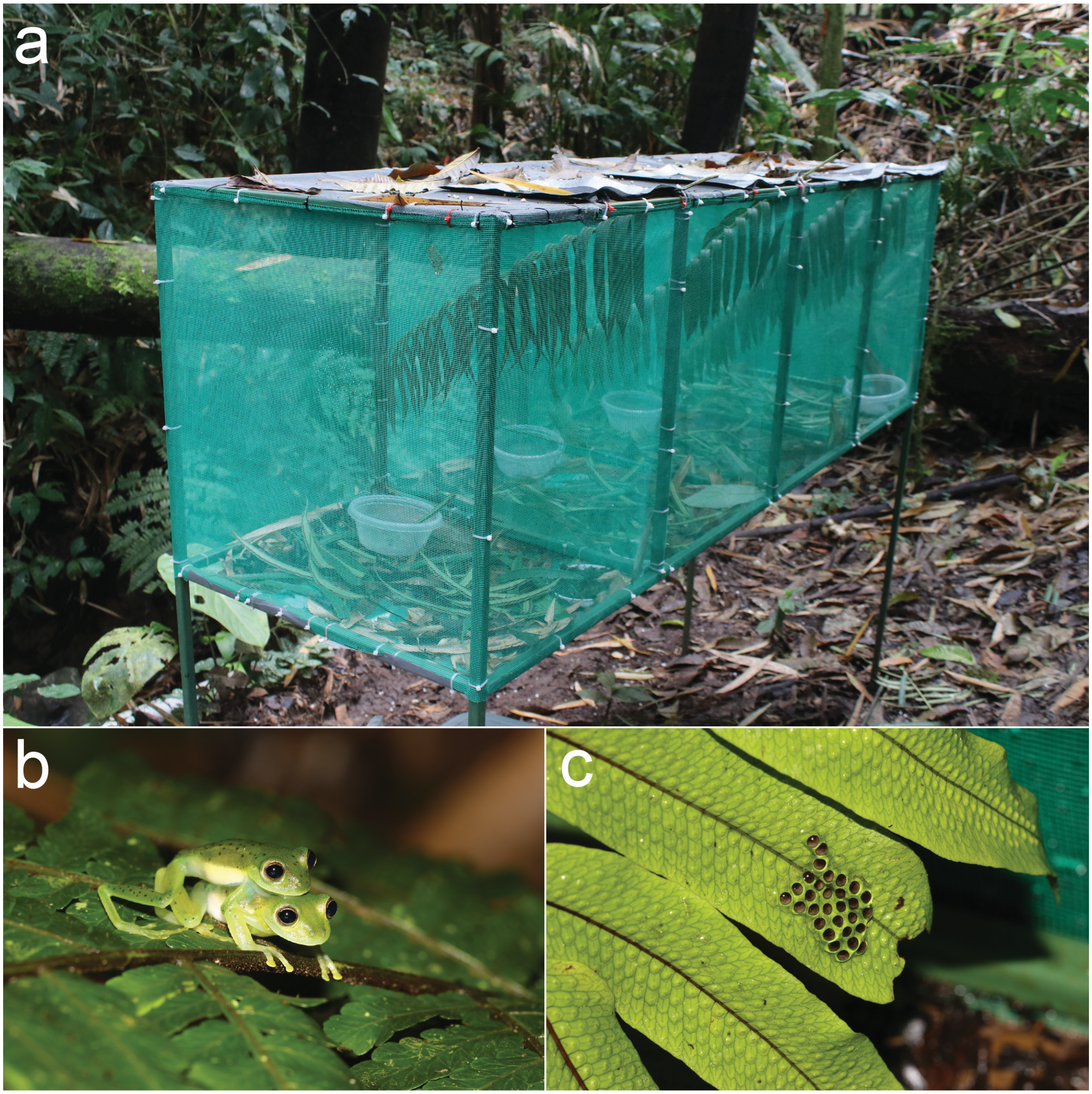

Supplemental Figure 1. (a) Setup of the semi-natural enclosure. The enclosure had four partitions adjacent to each other (38 x 50 x 75 cm) walled with green mesh. (b) A pair of *Espadarana prosoblepon* moving through the provided substrate inside the enclosure after capture and transportation. (c) A clutch of *E. prosoblepon*'s eggs laid on the provided substrate.
