# Supplementary material for: Influence of environmental factors and body condition on the post-oviposition behavior in the emerald glass frog Espadarana prosoblepon (Centrolenidae)
Source: PeerJ. 2022 Jun 16;10:e13616. doi: 10.7717/peerj.13616 (PMC9206843; doi:10.7717/peerj.13616)
Supplement: Supplemental Information 2 — Summary of model comparison and Akaike information criterion (AICc) values and Δ AICc values comparing different models examining the effect of environmental variables (temperature, humidity, mean wind speed, rainfall) on Espadarana prosoblepon’s post-oviposition behavior (POQ), using “cage number” as a random variable in all models. [file peerj-10-13616-s002.pdf]

Table S1. Summary of model comparison and Akaike information criterion (AIC_c) values and ΔAIC_c values comparing different models examining the effect of environmental variables (temperature, humidity, mean wind speed, rainfall) on *Espadarana prosoblepon*'s post-oviposition behavior (POQ), using "cage number" as a random variable in all models.

	**Candidate models**	***k***	**AIC_c**	**ΔAIC_c**	**w_i**
1	POQ ~ rainfall + (1| cage number)	4	19.41	0.00	0.34
2	POQ ~ 1 + (1| cage number)	3	19.92	0.50	0.27
3	POQ ~ temperature * rainfall + (1| cage number)	6	21.67	2.26	0.11
4	POQ ~ temperature + (1| cage number)	4	21.72	2.30	0.11
5	POQ ~ humidity * rainfall + (1| cage number)	6	23.24	3.82	0.05
6	POQ ~ mean wind speed + (1| cage number)	4	23.24	3.83	0.05
7	POQ ~ humidity + (1| cage number)	4	23.41	3.99	0.05
8	POQ ~ mean wind speed * rainfall + (1| cage number)	6	26.76	7.34	0.01
9	POQ ~ humidity * temperature + (1| cage number)	6	27.15	7.73	0.01
10	POQ ~ mean wind speed * temperature + (1| cage number)	6	29.81	10.39	0.00
11	POQ ~ rainfall + mean wind speed + temperature + humidity + (1| cage number)	7	30.51	11.10	0.00
12	POQ ~ mean wind speed * humidity + (1| cage number)	6	31.70	12.28	0.00
