# Supplementary material for: Influence of environmental factors and body condition on the post-oviposition behavior in the emerald glass frog Espadarana prosoblepon (Centrolenidae)
Source: PeerJ. 2022 Jun 16;10:e13616. doi: 10.7717/peerj.13616 (PMC9206843; doi:10.7717/peerj.13616)
Supplement: Supplemental Information 3 — Comparison of reported values of female snout-vent length, amplexus duration, whether brooding behavior was observed, female coverage of the clutch, and the time females remained with their clutch (defined in this study as ”post-oviposition quiescence period”) across different studies. Table entries include “—” if not reported. [file peerj-10-13616-s003.pdf]

Table S1. Summary of reproductive and behavioral biology parameters in *E. prosoblepon*. Parameters include female snout-vent length, amplexus duration, whether brooding behavior was observed, female coverage of the clutch, and the time females remained with their clutch (defined in this study as "post-oviposition quiescence period"). Table entries include "—" if not reported.

Location	N	Female SVL (mm)	Amplexus duration (h)	Brooding behavior?	Clutch coverage?	Post-oviposition quiescence period (min)	Reference
Monteverde, Costa Rica	9	—	2.9 ± 1.67 [1.25–5.35]	No	Five females partially covering the clutch; four covering the entire clutch	10–131	Jacobson 1985
Armenia, Colombia	9	29.88 ± 1.33 [27.54–31.84]	3.76 ± 1.74 [0.51–6.05]	—	Partially covering the clutch	45–174	Basto-Riascos et al.
—	7	—	—	Yes	—	80–97	Delia et al. 2017
San Vito, Costa Rica	20	23.3 ± 1.1 [21.2–25.0]	5.8 ± 1.4 [3.6–7.8]	No	Partially covering the clutch	22–158	This study
